# Selection of Single-Domain Antibodies towards Western Equine Encephalitis Virus

**DOI:** 10.3390/antib7040044

**Published:** 2018-12-15

**Authors:** Jinny L. Liu, Lisa C. Shriver-Lake, Dan Zabetakis, Ellen R. Goldman, George P. Anderson

**Affiliations:** Naval Research Laboratory, Center for Biomolecular Science and Engineering, Washington, DC 20375, USA; jinny.liu@nrl.navy.mil (J.L.L.); lisa.shriverlake@nrl.navy.mil (L.C.S.-L.); daniel.zabetakis@nrl.navy.mil (D.Z.); ellen.goldman@nrl.navy.mil (E.R.G.)

**Keywords:** single-domain antibody, Western equine encephalitis virus, MagPlex

## Abstract

In this work, we describe the selection and characterization of single-domain antibodies (sdAb) towards the E2/E3E2 envelope protein of the Western equine encephalitis virus (WEEV). Our purpose was to identify novel recognition elements which could be used for the detection, diagnosis, and perhaps treatment of western equine encephalitis (WEE). To achieve this goal, we prepared an immune phage display library derived from the peripheral blood lymphocytes of a llama that had been immunized with an equine vaccine that includes killed WEEV (West Nile Innovator + VEWT). This library was panned against recombinant envelope (E2/E3E2) protein from WEEV, and seven representative sdAb from the five identified sequence families were characterized. The specificity, affinity, and melting point of each sdAb was determined, and their ability to detect the recombinant protein in a MagPlex sandwich immunoassay was confirmed. Thus, these new binders represent novel recognition elements for the E2/E3E2 proteins of WEEV that are available to the research community for further investigation into their applicability for use in the diagnosis or treatment of WEE.

## 1. Introduction

Western equine encephalitis virus (WEEV), an alphavirus in the Togaviridae family, is an arbovirus transmitted to people and horses by mosquitoes and is the causative agent of western equine encephalitis (WEE). WEEV originated when Eastern equine encephalitis virus (EEEV), a new world virus, and Sindbis virus, an old world virus, recombined [1,2,3]. WEEV, EEEV and the related Venezuelan equine encephalitis virus (VEEV) can spread to the central nervous system, causing symptoms ranging from mild febrile reactions to encephalitis, often resulting in permanent neurological damage that can lead to death. In North America, WEE is seen primarily in U.S. States and Canadian provinces west of the Mississippi River [4]. The disease is also seen in South and Central American countries. WEEV causes serious disease in horses, with a case-fatality rate of 20–30% [5]. Although human cases are relatively rare, in 1941 an outbreak in Canada caused more than 1000 human infections [6]. For humans, the WEEV case-fatality rate has been estimated at 3 to 7%, with 15 to 30% of convalescent patients developing secondary neurological damage [7,8]. In addition to central nervous system impairment, demyelination is a known sequela of this disease. Additional complications can include mental retardation, behavioral changes, paralysis, permanent focal neurologic deficits, seizure disorders, cerebellar damage, and choreoathetosis. 

Alphaviruses can be produced in large quantities, are easy to disseminate and are highly infectious aerosols [9,10]; thus, WEEV, as well as VEEV and EEEV, are considered to be potential biological weapons [11,12,13] and are classified as category B bioterrorism agents/diseases by the US Centers for Disease Control and Prevention (https://emergency.cdc.gov/agent/agentlist-category.asp).

WEEV, like other alphaviruses, is an enveloped, positive-stranded RNA virus. Two envelope proteins, glycoproteins E1 and E2, associate as trimers of E1-E2 dimers on the viral surface, and make up 80 spikes on the viral surface. Structural studies of alphaviruses including cryo-EM of WEEV have been reported [14]. The E3 protein binds the E1-E2 spike, and protects it from the low pH of the secretory pathway [15]. 

There are available equine vaccines for WEEV which are recommended for the majority of horses. The vaccines typically contain inactivated virus and an adjuvant. There is currently no vaccine to protect humans from WEEV, however various vaccination strategies have been investigated. Due to the virus’s broad geographic distribution but low levels of infection accompanied with serious complications, an active human vaccination program is unlikely even if an effective vaccine were available. Nonetheless, rapid and inexpensive detection methodologies are still needed, and once identified, post-exposure therapies to reduce the risk of complications would be beneficial. A number of antibodies have been developed, including two antibody fragments (scFv) generated from murine IgGs for diagnostic purposes [16,17,18]. To date, the most extensively studied antibodies are scFv-Fc recombinant antibodies selected via phage display from two macaques immunized with inactivated WEEV. Several of these antibodies were found to be neutralizing [19] and in a later study were found to protect mice from an aerosol challenge [20]. While much progress has been made, there is clearly a need for additional reagents that possess properties different than those currently being investigated.

Single-domain antibodies (sdAb) are small and stable binding domains derived from the variable domain of the heavy-chain-only antibodies (termed VHH) found in camelids including camels, llamas, and alpacas. SdAb combine the sensitivity and specificity of conventional antibodies with advantages that come from being comprised of only a single domain, such as high physical-chemical stability including heat-resistance, the ability to refold after denaturation, excellent solubility in water, and the capacity to be produced using recombinant technology in good yield [21,22,23,24,25,26]. SdAb that are produced using recombinant technology, most often in *Escherichia coli*, are amenable to the formation of fusion constructs to tailor their integration into a variety of assay formats and sensor systems [27,28,29,30,31,32]. They can also be modified to improve their biophysical properties; mutagenesis has led to variants with improved protein production and stability, as assessed by the protein’s melting point [33,34,35,36,37]. In addition, one can take what is already a rugged and reliable immunoreagent and create even more robust versions for detection applications in resource-limited areas that lack refrigeration. Challenges remain for transitioning sdAb as therapeutics including rapid clearance from circulation due to their small size [38], and the presence of anti-domain antibodies in many individuals [39]. In this work, we describe the selection and characterization of seven new sdAb towards the E2/E3E2 envelope protein of WEEV. 

## 2. Materials and Methods

### 2.1. Reagents

Unless otherwise specified, chemical reagents were from Sigma Aldrich (St. Louis, MO, USA), Thermo Fisher Scientific (Waltham, MA, USA), or VWR International (Radnor, PA, USA). Restriction endonucleases and ligation reagents were from New England Biolabs (Ipswich, MA, USA). Recombinant WEEV glycoprotein (E2/E3E2) was purchased from IBT Bioservices (Rockville, MD, USA). The data in the product insert indicated that the majority of the preparation is E2, however E3E2 was also observed by both gel electrophoresis and Western blotting. For simplicity, we refer to this product as E3E2. The West Nile innovator + VEWT equine vaccine is produced by Zoetis (Parsippany-Troy Hills, NJ, USA) and is available from numerous US veterinary supply outlets.

A number of recombinantly produced proteins and virus-like particles (VLPs) were used to assess the specificity of selected binders. Chikungunya virus (CHIKV) E2 protein, E1 protein, and VLPs were from The Native Antigen Company (UK); EEEV E2/E3E2 was from IBT Bioservices; West Nile virus envelope protein was from Prospec (Israel); Lassa virus VLPs were from Zalgen (Germantown, MD, USA). 

### 2.2. Library Construction, Panning, and Production of sdAb 

Llama immunizations were through Triple J Farms, Bellingham, WA. Peripheral blood lymphocytes were isolated from a llama immunized with West Nile Innovator + VEWT, an equine vaccine that includes killed WEEV. Starting from these cells, we prepared total RNA, produced cDNA, amplified the coding sequence from the variable heavy domains, and constructed a phage display library as described previously [40]. Three rounds of panning, using the E3E2 protein adsorbed to wells of 96-well plates, were carried out essentially as previously described [40]. Positive clones were identified by a combination of monoclonal phage ELISA and monoclonal phage MagPlex assay after the second and third rounds [41].

The coding sequences for the sdAb were each mobilized from the pecan21 [42] phage display vector into pET22b as *NcoI-NotI* fragments as described previously [43]. In two cases, the sequence of the identified clone had an amber stop codon. In these cases, oligos were purchased to revert the amber stop codon to glutamine [44], and the clone is indicated by the suffix “f”. The sdAb expression plasmids were transformed into Tuner (DE3) for protein production. Freshly transformed colonies were used to start overnight cultures in 50 mL terrific broth (TB) containing ampicillin (100 µg/mL) at 25 °C. The next day, the overnight cultures were poured into 450 mL of TB with ampicillin and grown for 2 h at 25 °C prior to induction with isopropyl-D-1 thiogalactoside (IPTG, 0.5 mM) and a further 2 h growth.

**Figure A1:**
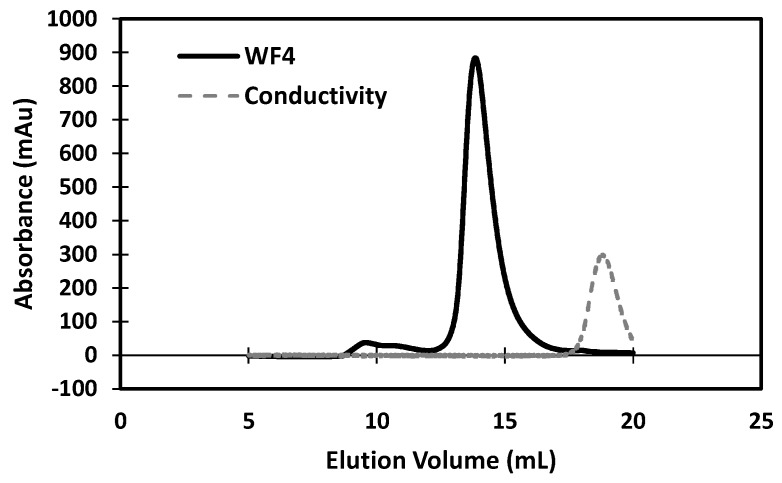
Representative gel-filtration chromatography trace.

Purification of sdAb expressed from pET22b, the periplasmic expression vector, was carried out through an osmotic shock protocol as described previously [45], followed by immobilized metal affinity chromatography (IMAC) resin (Ni Sepharose High Performance, GE Healthcare, Marlborough, MA, USA) eluted with 0.25 M imidazole prior to purification by fast protein liquid chromatography (FPLC) on a Enrich SEC 70 (10 × 300 mm) column (Bio-Rad, Hercules, CA, USA) equilibrated with phosphate buffered saline (PBS) with 0.02% sodium azide. Typical FPLC results are shown in Appendix A Figure A1. All sdAb eluted nearly entirely as monomers; only minor contaminants were left to be removed by gel filtration following IMAC chromatography. Only the monomeric fraction was used for further characterization. SdAb concentration was determined by UV absorption and stored at 4 °C or at −80 °C for long-term storage.

### 2.3. Surface Plasmon Resonance

Affinity and kinetics measurements were performed using the ProteOn XPR36 (Bio-Rad, Hercules, CA, USA). Two lanes of a general layer compact (GLC) chip were individually coated with WEEV E3E2 His tagged recombinant protein from IBT Bioservices diluted to 20 µg/mL in 10 mM sodium acetate pH 5.0, as described previously [46]. The other four lanes were left uncoated for this work. The binding affinity of each sdAb was determined by doing what is referred to as “One-Shot kinetics”, wherein each sdAb is flowed over the chip at a range of five concentrations and a blank to rapidly provide an array of binding curves. The chip is then regenerated by brief exposure to 0.085% phosphoric acid, then the next sdAb is analyzed. Data analysis was performed with ProteOn Manager 2.1 software, corrected by subtraction of the zero-antibody concentration column as well as interspot correction. The standard error of the fits was less than 10%. Binding constants were determined using the Langmuir model or the Langmuir with Mass Transfer built into the analysis software with the average of two determinations reported.

### 2.4. Determining Melting Temperature by Fluorescent Dye Melt Assay

The fluorescent dye melt assay which allows us to assess the inherent thermal stability of each sdAb was performed as described previously [47]. Each sdAb was first diluted to a concentration of 500 µg/mL in a final volume of 20 µL PBS. Next, a 1:1000 dilution of Sypro Orange dye (Sigma Aldrich) was added to each sample. Samples were measured in triplicate using a Step One Real-Time polymerase chain reaction (PCR) machine (Applied Biosystems, Foster City, CA, USA). The heating program was run in continuous mode from 25 °C–99 °C at a heating rate of 1% (~2 °C per minute), and data was recorded using the ROX filter. The melting point was determined to be the peak of the first derivative of the fluorescence intensity.

### 2.5. MagPlex Direct Binding and Sandwich Assays

Specificity was evaluated via direct binding to WEEV E3E2 recombinant protein immobilized on MagPlex magnetic microspheres (Luminex, Austin, TX, USA). The WEEV E3E2 along with a number of other viral proteins and VLPs in PBS (pH 7.2), as listed in 2.1, were immobilized to different sets of MagPlex microspheres using the standard two-step 1-ethyl-3-(3-dimethylaminopropyl)carbodiimide hydrochloride /N-hydroxysulfosuccinimide (Thermo Fisher Scientific) immobilization protocol recommended by the manufacture. Each of the sdAb was biotinylated using a 10-fold excess of EZ-Link NHS-LC-LC-Biotin (Thermo Fisher Scientific) for 30 min and then the excess biotin was removed using Zeba spin columns (Thermo Fisher Scientific) with the sdAb concentration determined by absorbance at 280 nM. Dilutions of each biotinylated sdAb (Bt-sdAb) in PBSTB (PBS + 0.05% Tween + 0.1% BSA) were prepared in round bottom polypropylene microtiter plates (VWR). To each was added the mixture of antigen-coated MagPlex microspheres sufficient to provide counts of least 50 for each set per well. After an incubation of 30 min, the plate was washed twice with PBST and then incubated with 5 µg/mL streptavidin-conjugated phycoerythrin (Thermo Fisher Scientific) for 30 min, washed, and binding evaluated on the MAGPIX instrument (Luminex).

Sandwich format MagPlex bead assays were performed in order to demonstrate the ability of the sdAb to act as both the capture and recognition reagent for the detection of WEEV E3E2 protein. For this assay, each sdAb was immobilized to a set of MagPlex microspheres as described above, and added to dilutions of WEEV E3E2 in PBSTB also as above. Then, each of the Bt-sdAb was utilized as the recognition molecule.

## 3. Results

**Figure 1:**
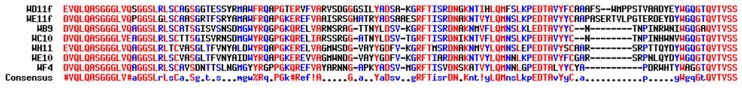
Deduced protein sequences of the seven single-domain antibodies (sdAb) that were evaluated. Sequences are given in single letter amino acid code. Alignment was performed using Multalin; high homology positions are shown in red, where lower homology is in blue [48].

Following immunization of a llama with the West Nile Innovator + VEWT equine vaccine, a phage display library was prepared that contained as much of the VHH immune repertoire of the animal as possible. After three rounds of panning on WEEV E3E2 recombinant protein immobilized on microtiter plates followed by evaluation of monoclonal phage from the second and third rounds by ELISA and MagPlex, 24 potential binding clones were identified. Four positive clones were identified from round 2 out of 32 screened, while 20 out of 64 round 3 clones had signal at least twice the background and were considered positive. Following sequencing of the identified potential binding clones, it was determined that the isolated clones segregated into five different sequence families based on homology of their CDR 3 sequence. Every sequence family except the one typified by WF4 had at least two different members. At least one representative clone was chosen from each family for further characterization (Figure 1). 

Each of the selected sequences was then cloned into an expression vector (pET22b) and produced via *E. coli* in 0.5 liter-scale shake flasks and purified. Protein yields are shown in Table 1. The majority of produced protein was monomeric with very little potential aggregation observed; a typical FPLC chromatogram is shown in Appendix A Figure A1. With the exception of WF4, all produced more than 10 mg/L; WE10 had the best yield at ~55 mg/L. The melting temperature (Tm) of each clone was measured using a fluorescent dye melt assay to evaluate their innate utility for high temperature applications, Table 1. Clone WF4 was least stable with a Tm of 43 °C, while WE10 was the most stable, melting at 75 °C. In general, however, no correlation between yield and Tm was observed.

**Figure A2:**
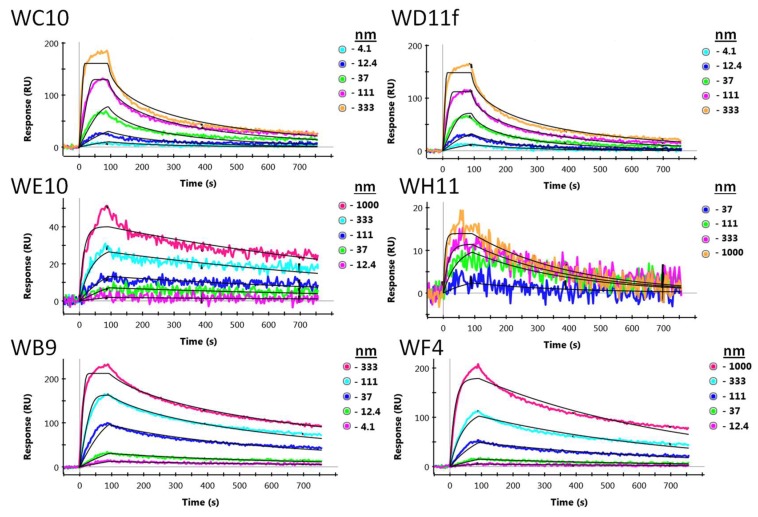
Representative surface plasmon resonance data.

The affinity of each of the sdAb for the WEEV E3E2 recombinant protein was determined by SPR; results also shown in Table 1. Representative SPR plots are shown in Appendix A Figure A2. The sdAb showed a wide range of on rates as well as off rates, however they resulted in dissociation constants that varied between 2.6 and 24 nM. Only one of the sdAb, WE11f, failed to show binding via SPR. While it could be that its epitope was obscured during immobilization of the E3E2 recombinant protein to the sensor chip, based on the direct binding results presented below, it is clear that it was at best a relatively weak binder. 

**Figure 2:**
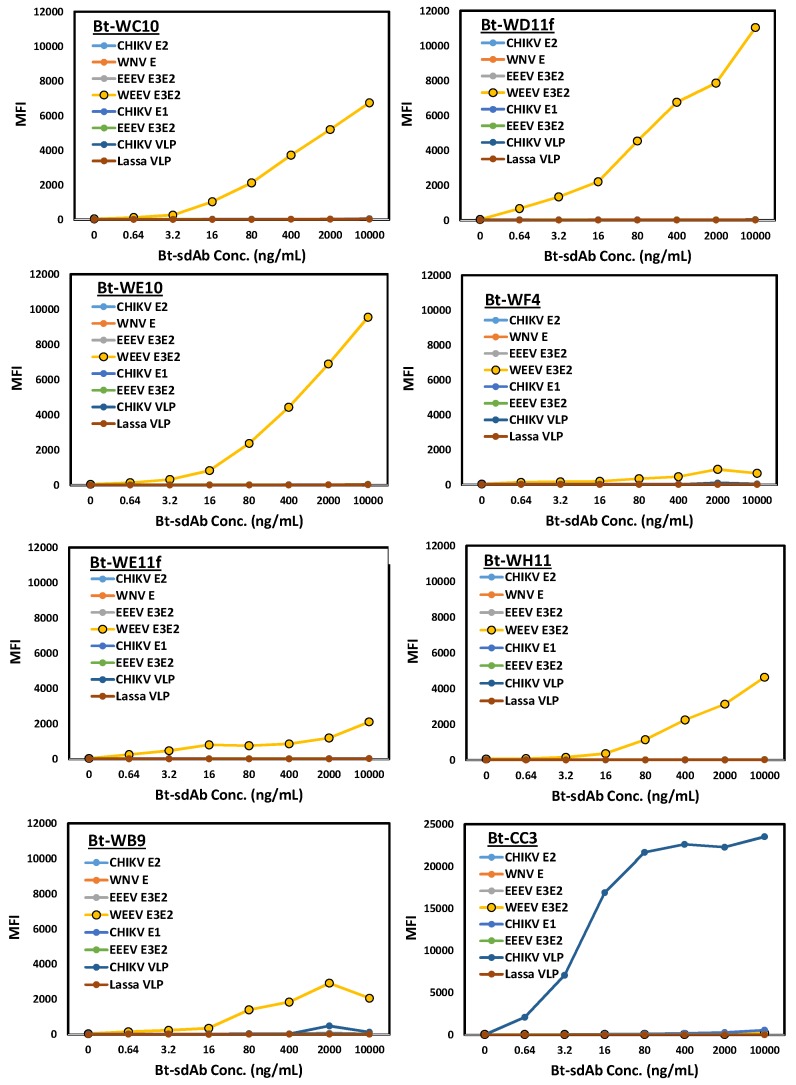
MagPlex direct binding assay to evaluate the specificity of the putative anti-WEEV E3E2 binding sdAb. Each sdAb was biotin labeled and mixed with MagPlex microspheres coated with various antigens at a range of concentrations. Biotinylated sdAb are denoted by Bt-clone name. Median fluorescence intensity versus Bt-sdAb concentration is plotted. The anti CHIKV sdAb CC3 was also tested as a positive control for the activity of the other sets of microspheres [49].

The MagPlex direct binding results, shown in Figure 2, do not show as much correlation with the binding affinities determined by SPR as one might expect. The three best binders in the MagPlex assay were WC10, WD11f, and WE10. While one did not expect much of WE11f, as it failed to bind via the SPR, the other three possess affinities as good as or better than the three which displayed the best binding in this assay. The two assays, SPR and MagPlex, have important differences, which can easily lead to a lack of correlation; the SPR sensor chips and MagPlex microspheres both use a similar chemistry for protein immobilization, but the reaction is done at different pHs, pH 5.0 for SPR and pH 7.2 for MagPlex, which can impact the orientation of antigen attachment. Even more critically, the sdAb is biotinylated for the MagPlex assays which can negatively impact the activity of the binder. Nonetheless, the most important aspect of this assay was that all the selected binding sdAb showed good specificity, having virtually no binding to any other antigen evaluated. The anti-CHIKV sdAb CC3, tested primarily to demonstrate the activity of at least one of the specificity controls, showed better binding activity than the selected WEEV binders likely due to its superior affinity for CHIKV VLP (ka: 5.2 × 10^5^; kd: 3.3 × 10^−4^, KD: 6.5 × 10^−10^) [49].

**Figure 3:**
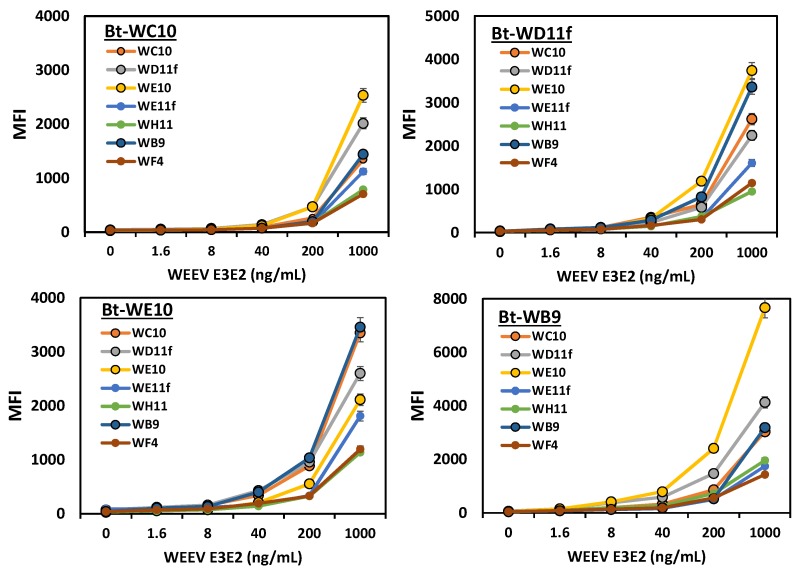
MagPlex sandwich fluoroimmunoassay to evaluate the utility of the sdAb to function as both a capture and recognition reagent for the detection of WEEV E3E2 protein. Only the four best Bt-sdAb recognition reagents are shown. Median fluorescence intensity versus WEEV E3E2 concentration is plotted; error bars represent the SEM.

The final experiment looked at using the anti-WEEV sdAb as both capture and biotinylated recognition reagents in a MagPlex sandwich fluoroimmunoassay. The four best biotinylated recognition elements paired with each of the capture bead sets are shown in Figure 3. Not surprisingly, three of the four best biotinylated recognition sdAb were WC10, WD11f, and WE10, as those three also performed best in the MagPlex direct binding assay. WB9 also performed well in this assay. While not doing well in the direct binding assay, WB9 has a sequence very similar to WC10 and had the lowest dissociation constant (2.6 nM), thus it was not surprising that it did well. These same four sdAb were also the four best capture sdAb. Since the same sdAb could function as both the capture and biotinylated recognition reagent, it can be assumed that the E3E2 protein is at least partially aggregated, however the best capture for each biotinylated recognition sdAb was one of the other three, so it would appear a sufficient amount of the WEEV E3E2 protein was monomeric to make the use of separate epitopes for the capture and recognition reagents beneficial. 

## 4. Discussion

This work describes the initial isolation and characterization of anti-WEEV E2/E3E2 sdAb. Several antibodies have now been described that are useful for the detection and perhaps the treatment of WEEV [19,20]. We selected sdAb for binding to WEEV envelope protein, specifically E2 and E3E2. This is a promising target, as several neutralizing epitopes have been found on the E2 of the related VEEV [50]. There is also evidence that suggests antibodies directed against E3 can provide protection against alphaviruses in mice [51]. 

SdAb offer an alternative to conventional antibodies and their derivatives that can be easily tailored to possess additional functionalities as desired. For instance, the sdAb isolated here all have affinities between ~2.6 and 24 nM, whereas to obtain adequate sensitivity or activity one may desire sub-nM apparent affinities. This can easily be achieved for sdAb by the formation of multimer constructs that can take advantage of avidity. For viral targets that are naturally multimeric, this is a valid approach to increase the apparent binding affinity. These types of constructs have been demonstrated by many groups including ourselves [52,53,54]. Additionally, several strategies have been described for increasing the stability of sdAb [37], and have been shown to increase melting temperatures by as much as 20 °C [43]. Similarly, sdAb can be engineered to tailor them for specific assay formats [28,32,55].

There are many examples of viral neutralizing sdAb [56,57]; expressing the sdAb as genetic fusions has led to improved neutralization [58,59,60]. In one example, viral neutralizing sdAb have been generated through selections against the trimeric envelope proteins of Respiratory Syncytial Virus, Rabies virus and H5N1 Influenza. In that work, the researchers identified neutralizing sdAb recognizing different epitopes in the receptor binding sites on the spikes with affinities in the low nanomolar range. Multimeric constructs, in which the sdAb were genetically linked improved neutralization potencies up to 4000-fold for RSV, 1500-fold for Rabies virus and 75-fold for Influenza H5N1 and had potencies similar to or better than the best performing monoclonal antibodies [59]. The trivalent sdAb construct (ALX-0171) that inhibits RSV has also been successfully delivered by inhalation [61], a route that may also prove valuable for treatment of alphaviruses that are transmitted via aerosols.

It is probable that additional binders for WEEV E2 and E3E2 exist in our constructed library. This was only our first look at this library and if additional binders are desired, a greater number of clones can be evaluated or additional rounds of panning can easily be performed. If these anti-WEEV binding sdAb are proven to be neutralizing and protective, they may make for attractive alternatives to the larger antibody fragments, as their robust nature may allow them to be stored without refrigeration making them ideal for use in resource-limited areas that lack the power grid infrastructure and where the mosquito transmission of these arbovirus is widespread. 

## Figures and Tables

**Table 1:** Anti-WEEV sdAb yields, melting points, and binding affinities.

Clone	Yield (mg/L)	Tm °C	ka (1/Ms)	kd (1/s)	KD (M)
WC10	48	51	1.0 × 10^8^	8.5 × 10^−1^	8.5 ×10^−9^
WD11f	18	50	7.3 × 10^7^	5.3 × 10^−1^	7.2 ×10^−9^
WE10	55	75	7.0 × 10^4^	8.3 ×10^−4^	1.2 × 10^−8^
WE11f	38	60	nbo	nbo	nbo
WH11	15	72	1.5 × 10^5^	3.3 × 10^−3^	2.2 × 10^−8^
WB9	21	54	1.1 × 10^6^	2.9 × 10^−3^	2.6 × 10^−9^
WF4	3	43	6.3 × 10^4^	1.5 × 10^−3^	2.4 × 10^−8^

nbo indicates no binding observed.

## References

[1] Hahn C.S., Lustig S., Strauss E.G., Strauss J.H. (1988). Western equine encephalitis virus is a recombinant virus. Proc. Natl. Acad. Sci. USA.

[2] Powers A.M., Brault A.C., Shirako Y., Strauss E.G., Kang W., Strauss J.H., Weaver S.C. (2001). Evolutionary relationships and systematics of the alphaviruses. J. Virol..

[3] Strauss J.H., Strauss E.G. (1994). The alphaviruses: Gene expression, replication, and evolution. Microb. Rev..

[4] Goh L.Y., Hobson-Peters J., Prow N.A., Gardner J., Bielefeldt-Ohmann H., Pyke A.T., Suhrbier A., Hall R.A. (2013). Neutralizing monoclonal antibodies to the e2 protein of chikungunya virus protects against disease in a mouse model. Clin. Immunol..

[5] Eastern, Western and Venezuelan Equine Encephalomyelitis. http://www.cfsph.iastate.edu/Factsheets/pdfs/easter_wester_venezuelan_equine_encephalomyelitis.pdf.

[6] Reisen W., Monath T., Monath T.P. (1989). Western equine encephalitis. The Arboviruses: Epidemiology and Ecology.

[7] Zacks M.A., Paessler S. (2010). Encephalitic alphaviruses. Vet. Microbiol..

[8] Reeves W.C., Hutson G.A., Bellamy R.E., Scrivani R.P. (1958). Chronic latent infections of birds with western equine encephalomyelitis virus. Proc. Soc. Exp. Biol. Med..

[9] Phillpotts R.J. (2006). Venezuelan equine encephalitis virus complex-specific monoclonal antibody provides broad protection, in murine models, against airborne challenge with viruses from serogroups i, ii and iii. Virus Res..

[10] Reed D.S., Larsen T., Sullivan L.J., Lind C.M., Lackemeyer M.G., Pratt W.D., Parker M.D. (2005). Aerosol exposure to western equine encephalitis virus causes fever and encephalitis in cynomolgus macaques. J. Infect. Dis..

[11] Hawley R.J., Eitzen E.M. (2001). Biological weapons—A primer for microbiologists. Annu. Rev. Microb..

[12] Sidwell R.W., Smee D.F. (2003). Viruses of the bunya- and togaviridae families: Potential as bioterrorism agents and means of control. Antiv. Res..

[13] Nagata L.P., Wong J.P., Hu W.-G., Wu J.Q. (2013). Vaccines and therapeutics for the encephalitic alphaviruses. Future Virol..

[14] Sherman M.B., Weaver S.C. (2010). Structure of the recombinant alphavirus western equine encephalitis virus revealed by cryoelectron microscopy. J. Virol..

[15] Uchime O., Fields W., Kielian M. (2013). The role of e3 in ph protection during alphavirus assembly and exit. J. Virol..

[16] Das D., Kriangkum J., Nagata L.P., Fulton R.E., Suresh M.R. (2004). Development of a biotin mimic tagged scfv antibody against western equine encephalitis virus: Bacterial expression and refolding. J. Virol. Methods.

[17] Xu B., Kriangkum J., Nagata L.P., Fulton R.E., Suresh M.R. (1999). A single chain fv specific against western equine encephalitis virus. Hybridoma.

[18] Long M.C., Jager S., Mah D.C.W., Jebailey L., Mah M.A., Masri S.A., Nagata L.P. (2000). Construction and characterization of a novel recombinant single-chain variable fragment antibody against western equine encephalitis virus. Hybridoma.

[19] Hülseweh B., Rülker T., Pelat T., Langermann C., Frenzel A., Schirrmann T., Dübel S., Thullier P., Hust M. (2014). Human-like antibodies neutralizing western equine encephalitis virus. mAbs.

[20] Burke C.W., Froude J.W., Miethe S., Hülseweh B., Hust M., Glass P.J. (2018). Human-like neutralizing antibodies protect mice from aerosol exposure with western equine encephalitis virus. Viruses.

[21] Hamers-Casterman C., Atarhouch T., Muyldermans S., Robinson G., Hamers C., Songa E.B., Bendahman N., Hamers R. (1993). Naturally occurring antibodies devoid of light chains. Nature.

[22] Ghahroudi M.A., Desmyter A., Wyns L., Hamers R., Muyldermans S. (1997). Selection and identification of single domain antibody fragments from camel heavy-chain antibodies. FEBS Lett..

[23] De Marco A. (2011). Biotechnological applications of recombinant single-domain antibody fragments. Microb. Cell Fact..

[24] Muyldermans S. (2013). Nanobodies: Natural single-domain antibodies. Annu. Rev. Biochem..

[25] Wesolowski J., Alzogaray V., Reyelt J., Unger M., Juarez K., Urrutia M., Cauerhff A., Danquah W., Rissiek B., Scheuplein F. (2009). Single domain antibodies: Promising experimental and therapeutic tools in infection and immunity. Med. Microbiol. Immunol..

[26] Eyer L., Hruska K. (2012). Single-domain antibody fragments derived from heavy-chain antibodies: A review. Vet. Med..

[27] Hussack G., Hirama T., Ding W., MacKenzie R., Tanha J. (2011). Engineered single-domain antibodies with high protease resistance and thermal stability. PLoS ONE.

[28] Liu J.L., Walper S.A., Turner K.B., Brozozog-Lee A., Medintz I.L., Susumu K., Oh E., Zabetakis D., Goldman E.R., Anderson G.P. (2016). Conjugation of biotin-coated luminescent quantum dots with single domain antibody-rhizavidin fusions. Biotechnol. Rep..

[29] Liu J.L., Zabetakis D., Brozozog Lee P.A., Goldman E.R., Anderson G.P. (2013). Single domain antibody alkaline phosphatase fusion proteins for antigen detection—Analysis of affinity and thermal stability of single domain antibody. J. Immunol. Methods.

[30] Pleschberger M., Saerens D., Weigert S., Sleytr U.B., Muyldermans S., Sara M., Egelseer E.M. (2004). An s-layer heavy chain camel antibody fusion protein for generation of a nanopatterned sensing layer to detect the prostate-specific antigen by surface plasmon resonance technology. Bioconjug. Chem..

[31] Raphael M.P., Christodoulides J.A., Byers J.M., Anderson G.P., Liu J.L., Turner K.B., Goldman E.R., Delehanty J.B. (2015). Optimizing nanoplasmonic biosensor sensitivity with orientated single domain antibodies. Plasmonics.

[32] Sherwood L.J., Hayhurst A. (2012). Hapten mediated display and pairing of recombinant antibodies accelerates assay assembly for biothreat countermeasures. Sci. Rep..

[33] Liu J.L., Goldman E.R., Zabetakis D., Walper S.A., Turner K.B., Shriver-Lake L.C., Anderson G.P. (2015). Enhanced production of a single domain antibody with an engineered stabilizing extra disulfide bond. Microb. Cell Fact..

[34] Hagihara Y., Mine S., Uegaki K. (2007). Stabilization of an immunoglobulin fold domain by an engineered disulfide bond at the buried hydrophobic region. J. Biol. Chem..

[35] Saerens D., Conrath K., Govaert J., Muyldermans S. (2008). Disulfide bond introduction for general stabilization of immunoglobulin heavy-chain variable domains. J. Mol. Biol..

[36] Turner K.B., Liu J.L., Zabetakis D., Lee A.B., Anderson G.P., Goldman E.R. (2015). Improving the biophysical properties of anti-ricin single-domain antibodies. Biotechnol. Rep..

[37] Goldman E.R., Liu J.L., Zabetakis D., Anderson G.P. (2017). Enhancing stability of camelid and shark single domain antibodies: An overview. Front. Immunol..

[38] Hu Y., Liu C., Muyldermans S. (2017). Nanobody-based delivery systems for diagnosis and targeted tumor therapy. Front. Immunol..

[39] Cordy J.C., Morley P.J., Wright T.J., Birchler M.A., Lewis A.P., Emmins R., Chen Y.Z., Powley W.M., Bareille P.J., Wilson R. (2015). Specificity of human anti-variable heavy (vh) chain autoantibodies and impact on the design and clinical testing of a vh domain antibody antagonist of tumour necrosis factor-alpha receptor 1. Clin. Exp. Immunol..

[40] Liu J.L., Shriver-Lake L.C., Anderson G.P., Zabetakis D., Goldman E.R. (2017). Selection, characterization, and thermal stabilization of llama single domain antibodies towards ebola virus glycoprotein. Microb. Cell Fact..

[41] Anderson G., Matney R., Liu J., Hayhurst A., Goldman E. (2007). Multiplexed fluid array screening of phage displayed anti-ricin single domain antibodies for rapid assessment of specificity. Biotechniques.

[42] Goldman E., Anderson G., Liu J., Delehanty J., Sherwood L., Osborn L., Cummins L., Hayhurst A. (2006). Facile generation of heat-stable antiviral and antitoxin single domain antibodies from a semisynthetic llama library. Analyt. Chem..

[43] Walper S.A., Liu J.L., Zabetakis D., Anderson G.P., Goldman E.R. (2014). Development and evaluation of single domain antibodies for vaccinia and the l1 antigen. PLoS ONE.

[44] Marcus W.D., Lindsay S.M., Sierks M.R. (2006). Identification and repair of positive binding antibodies containing randomly generated amber codons from synthetic phage display libraries. Biotechnol. Prog..

[45] Shriver-Lake L.C., Zabetakis D., Goldman E.R., Anderson G.P. (2017). Evaluation of anti-botulinum neurotoxin single domain antibodies with additional optimization for improved production and stability. Toxicon.

[46] Walper S.A., Lee P.A.B., Anderson G.P., Goldman E.R. (2013). Selection and characterization of single domain antibodies specific for bacillus anthracis spore proteins. Antibodies.

[47] Liu J.L., Zabetakis D., Goldman E.R., Anderson G.P. (2013). Selection and evaluation of single domain antibodies toward ms2 phage and coat protein. Mol. Immunol..

[48] Corpet F. (1988). Multiple sequence alignment with hierarchical-clustering. Nucleic. Acids Res..

[49] Liu J.L., Shriver-Lake L.C., Zabetakis D., Anderson G.P., Goldman E.R. (2019). Selection and characterization of protective anti-chikungunya virus single domain antibodies. Mol. Immunol..

[50] Hunt A.R., Frederickson S., Maruyama T., Roehrig J.T., Blair C.D. (2010). The first human epitope map of the alphaviral e1 and e2 proteins reveals a new e2 epitope with significant virus neutralizing activity. PLOS Negl. Trop. Dis..

[51] Parker M.D., Buckley M.J., Melanson V.R., Glass P.J., Norwood D., Hart M.K. (2010). Antibody to the e3 glycoprotein protects mice against lethal venezuelan equine encephalitis virus infection. J. Virol..

[52] Liu J.L., Zabetakis D., Walper S.A., Goldman E.R., Anderson G.P. (2014). Bioconjugates of rhizavidin with single domain antibodies as bifunctional immunoreagents. J. Immunol. Methods.

[53] Walper S.A., Brozozog Lee P.A., Goldman E.R., Anderson G.P. (2013). Comparison of single domain antibody immobilization strategies evaluated by surface plasmon resonance. J. Immunol. Methods.

[54] Zhang J., Tanha J., Hirama T., Khieu N.H., To R., Tong-Sevinc H., Stone E., Brisson J.-R., Roger MacKenzie C. (2004). Pentamerization of single-domain antibodies from phage libraries: A novel strategy for the rapid generation of high-avidity antibody reagents. J. Mol. Biol..

[55] Anderson G., Shriver-Lake L., Walper S., Ashford L., Zabetakis D., Liu J., Breger J., Brozozog Lee P., Goldman E. (2018). Genetic fusion of an anti-bcla single-domain antibody with beta galactosidase. Antibodies.

[56] Vanlandschoot P., Stortelers C., Beirnaert E., Ibanez L.I., Schepens B., Depla E., Saelens X. (2011). Nanobodies(r): New ammunition to battle viruses. Antiviral. Res..

[57] Wu Y., Jiang S., Ying T. (2017). Single-domain antibodies as therapeutics against human viral diseases. Front. Immunol..

[58] Boruah B.M., Liu D., Ye D., Gu T.-J., Jiang C.-L., Qu M., Wright E., Wang W., He W., Liu C. (2013). Single domain antibody multimers confer protection against rabies infection. PLoS ONE.

[59] Hultberg A., Temperton N.J., Rosseels V., Koenders M., Gonzalez-Pajuelo M., Schepens B., Ibañez L.I., Vanlandschoot P., Schillemans J., Saunders M. (2011). Llama-derived single domain antibodies to build multivalent, superpotent and broadened neutralizing anti-viral molecules. PLoS ONE.

[60] Matz J., Kessler P., Bouchet J., Combes O., Ramos O.H., Barin F., Baty D., Martin L., Benichou S., Chames P. (2013). Straightforward selection of broadly neutralizing single-domain antibodies targeting the conserved cd4 and coreceptor binding sites of hiv-1 gp120. J. Virol..

[61] Larios Mora A., Detalle L., Gallup J.M., Van Geelen A., Stohr T., Duprez L., Ackermann M.R. (2018). Delivery of alx-0171 by inhalation greatly reduces respiratory syncytial virus disease in newborn lambs. mAbs.

